# Association between different metabolic phenotypes and the development of hypothyroidism: 9 years follow-up of Tehran thyroid study

**DOI:** 10.3389/fendo.2023.1134983

**Published:** 2023-03-10

**Authors:** Behnaz Abiri, Amirhossein Ramezani Ahmadi, Maryam Mahdavi, Farhad Hosseinpanah, Atieh Amouzegar, Majid Valizadeh

**Affiliations:** ^1^ Obesity Research Center, Research Institute for Endocrine Sciences, Shahid Beheshti University of Medical Sciences, Tehran, Iran; ^2^ Isfahan Endocrine and Metabolism Research Center, Isfahan University of Medical Sciences, Isfahan, Iran; ^3^ Endocrine Research Center, Research Institute for Endocrine Sciences, Shahid Beheshti University of Medical Sciences, Tehran, Iran

**Keywords:** metabolic phenotype, obesity phenotype, hypothyroidism, sex difference, follow-up

## Abstract

**Purpose:**

The association between metabolic phenotypes and thyroid function has not yet been established; therefore, this study examined whether different metabolic phenotypes are associated with the development of hypothyroidism.

**Methods:**

Study participants were selected from the Tehran Thyroid Study (TTS). A total of 3338 euthyroid adults were included and categorized into four obesity phenotype groups: metabolically healthy normal weight (MHNW), metabolically healthy obese (MHO), metabolically unhealthy normal weight (MUNW), and metabolically unhealthy obese (MUO). The participants were assessed at baseline and during three follow-up studies at three-year intervals. Multiple logistic regression analysis was used to examine the relationship between metabolic phenotypes and the development of hypothyroidism.

**Results:**

In the total population, the chi-square test was only significant (P=0.008) in 3^rd^ year with a higher prevalence of hypothyroidism in the MUNW phenotype, followed by MHO, MUO, and MHNW. Moreover, in the 3^rd^ and 9^th^ years of follow-up, hypothyroidism was more prevalent in MUO only in male subjects (P=0.002 and 0.035, respectively). In the unadjusted model, the MHO phenotype increased the odds of hypothyroidism compared with the MHNW phenotype (OR=1.51; 95% CI=1.04, 2.18; P-value=0.031). After adjusting for confounding factors, the odds of hypothyroidism were higher in the MUNW (OR=1.86; 95% CI=1.17, 2.96; P-value=0.008), MHO (OR=1.71; 95% CI=1.09, 2.67; P-value=0.018), and MUO (OR=1.64; 95% CI=1.03, 2.62; P-value=0.036) phenotypes than in the MHNW group. The MUNW phenotype increased the risk of hypothyroidism compared to MHNW, only in males. However, in females, the MHO phenotype increased the risk of hypothyroidism compared to MHNW.

**Conclusion:**

Both obesity and metabolic abnormalities are associated with hyperthyroidism. Healthy metabolic and weight maintenance were associated with a lower risk of hypothyroidism in males and females.

## 1 Introduction

Hypothyroidism is a common pathological condition characterized by a deficiency of thyroid hormones that can be overt or subclinical (1). There is limited information about the incidence of hypothyroidism in Middle Eastern countries. A systematic review (2) examined the prevalence of thyroid disease in ten Middle Eastern countries; however, the study population was heterogeneous. The incidence rates of subclinical and overt hypothyroidism in Tehran, the capital city of Iran, and an iodine-sufficient region, were 7.62 and 2.0 per 1000 individuals, respectively) (3). Evidence suggests that hypothyroidism increases the risk of cardiovascular events and mortality (4, 5). Considering these facts, identifying hypothyroidism risk factors is crucial for preventing its increase.

There is also a growing epidemic of obesity in the global population, with serious adverse health consequences. Recent studies have shown a link between obesity and thyroid dysfunction, and several studies have reported that obesity causes thyroid problems as well as being a result of them (6–8). According to a meta-analysis of 22 studies, obese individuals are more likely to have overt and subclinical hypothyroidism (9). Typically, obesity is linked to metabolic abnormalities including hypertension, hyperlipidemia, and hyperglycemia. According to a prospective cohort study, participants with metabolic syndrome at baseline are more likely to develop subclinical hypothyroidism in the future (10). Thus, both obesity and metabolic disorders are closely associated with hypothyroidism.

It is well known that obesity adversely affects metabolic health, but individual responses differ (11). Some individuals who are obese may have a metabolically healthy obese (MHO) phenotype (12). Furthermore, metabolically unhealthy normal weight (MUNW) refers to individuals who have abnormal metabolic parameters, but are not obese (13). It is more accurate to predict cardiovascular disease and mortality from obesity phenotypes that combine obesity with different metabolic profiles (14). Different types of obesity could also provide insight into whether obesity or coexisting metabolic abnormalities are associated with hypothyroidism.

Until now, the idea that thyroid function could be used to identify obesity phenotypes in individuals with euthyroidism has only been explored in a few studies (15). However, the relationship between metabolic phenotypes and thyroid function has not been determined. In the present study, we investigated the relationship between different metabolic phenotypes and the development of hypothyroidism, as well as the modulating effect of sex within a 9-year follow-up in a cohort of the Tehran Thyroid Study (TTS).

## 2 Methods and materials

### 2.1 Study population

The study participants were recruited from the TTS (16), a cohort study that is conducted within the framework of the Tehran Lipid and Glucose Study (TLGS). The TLGS is a long-term, ongoing community-based research to identify and prevent noncommunicable disorders being carried out in district No. 13, an area of about 13 km^2^, located in the eastern part of Tehran city, under coverage of Shahid Beheshti University of Medical Sciences and Health Services. In this area, three medical health centers with field data on more than 90% of all covered families were chosen. Baseline measurements were recorded and Three-year follow-up studies were conducted on participants. An initial sample of 15005 participants aged ≥ 3 years was selected by a multistage stratified cluster sampling method for the TLGS (17). Among 10368 subjects aged ≥20 years, 5786 participants who had thyroid function serum samples at baseline (February 1999– August 2001) and at all follow-up phases (up to March 2011) were chosen to include in the TTS.

**Figure 1:**
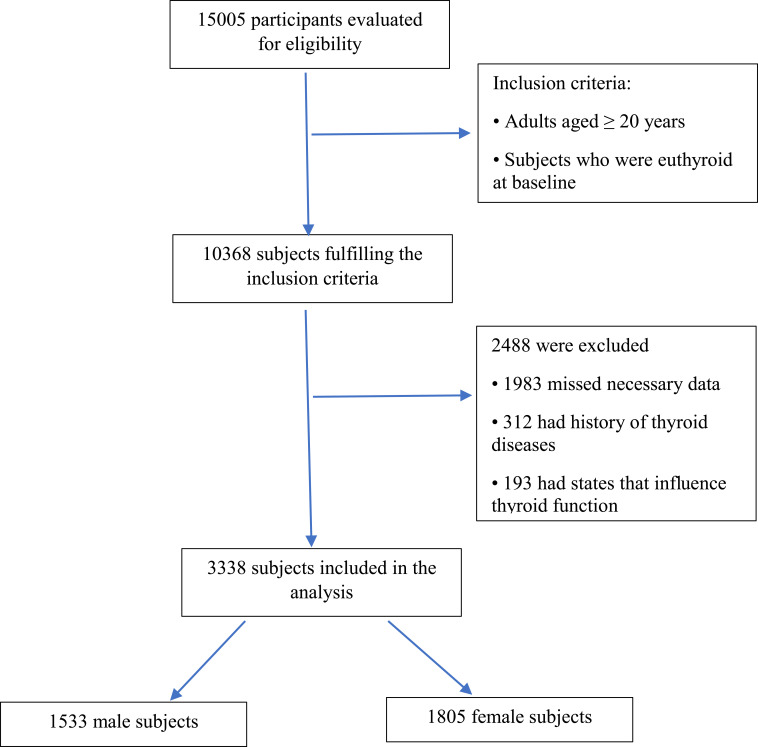
The flowchart of recruitment.

In the current study, the inclusion criteria were as follows: (1) adults aged ≥ 20; and (2) individuals with normal thyroid function at baseline. On the other hand, participants were excluded if they had a TSH <0.32 mIU/L or a TSH >5.06 mIU/L in any phase of the study (18). Patients with genetic disorders, addiction to alcohol and opium, and consumption of some effective drugs (important interfering factors that can impact other parameters). Those with levothyroxine, antithyroid drug, or corticosteroid usage, a history of thyroid surgery, thyroid radiation, or pregnant women were also excluded. Sixty-four individuals lacked data necessary to categorize obesity phenotypes. Finally, 3338 subjects were included. Ultimately, 1533 males and 1805 females were participated in our study (**Figure 1**). This study was approved by the ethics committee of Research Institute for Endocrine Sciences (RIES) of Shahid Beheshti University of Medical Sciences (code: IR.SBMU.ENDOCRINE.REC.1400.116). Written informed consent was obtained from all participants.

### 2.2 Anthropometric measurements

The participants who invited to the TTS were referred to trained physicians after signing an informed consent form. Participants wore light clothing and no shoes during the anthropometric measurements. Weight and height were determined using a digital electronic weighing scale (Seca 707; range 0.1–150 kg; Seca, Hanover, MD) with an accuracy of up to 100 g and a tape meter stadiometer, respectively. In order to calculate body mass index (BMI), weight (kg) was divided by height (meters) squared. We measured waist circumference (WC) in centimeters at the level of the umbilicus.

### 2.3 Measurements of metabolic indices

Blood samples were taken between 7:00 am and 9:00 am from all study participants, following an overnight fast of 12 to 14 hours. Fasting glucose levels were measured by glucose oxidase and enzymatic colorimetry. Serum total cholesterol (TC) and triglycerides (TGs) levels were determined using the enzymatic calorimetric method with cholesterol esterase, cholesterol oxidase, and glycerol phosphate oxidase, respectively. High-density lipoprotein cholesterol (HDL-C) was measured after the precipitation of apolipoprotein B-containing lipoproteins with phosphotungistic acid. All these biochemical tests were conducted on the day of sampling, using commercial kits (Pars Azmoon, Inc., Tehran, Iran) by the Selectra 2 auto-analyzer (Vital Scientific, Spankeren, The Netherlands). Analyses were performed on all the samples once quality control was achieved. Both inter- and intra-assay coefficients of variation (CVs) were <2.3% for glucose, <2% for TC, <2.1% for TG, and <3% for HDL-C.

fT4 and TSH concentrations were estimated in -70°C stored serum samples by the electrochemiluminescence immunoassay method using Roche Diagnostics kits and a Roche/Hitachi Cobas e-411 analyzer (Mannheim, Germany). Lyophilized quality control material (Lyphochek Immunoassay plus Control; Bio-Rad Laboratories, Hercules, CA) was used to monitor the accuracy of the assay. The intra- and inter-assay CVs were 1.3% and 3.7% for fT4 and 1.5% and 4.5% for TSH measurements, respectively. Thyroid peroxidase antibodies (TPOAb) were assayed by an immunoenzymometric assay kit (IEMA; Monobind, Costa Mesa, CA) and the Sunrise ELISA reader (Tecan Co., Salzburg, Austria); intra- and inter-assay CVs were 3.9% and 4.7%, respectively. In the RIES research laboratory, all measurements were performed simultaneously.

Once the subjects had rested for 15 minutes, a qualified physician measured their systolic blood pressure (SBP) and diastolic blood pressure (DBP) twice in a seated position. The first measurement was used to determine the peak inflation level using a mercury sphygmomanometer. In this study, participant’s blood pressure was calculated as the average of two measurements.

### 2.4 Definition of variables and outcomes

The reference ranges were 0.32–5.06 mIU/L for TSH, and 0.91-1.55 pmol/L for FT4. The reference range for serum TSH and FT4 levels was defined as euthyroidism. Hypothyroidism was defined as TSH > 5.06 mIU/L and FT4 < 0.91 pmol/L (overt hypothyroidism) or TSH > 5.06 mIU/L and FT4 levels within the reference range (subclinical hypothyroidism).

Using a BMI ≥ 25 kg/m^2^ as a threshold to define overweight/obesity seems to be a more reasonable approach (19). Abnormal metabolic components were defined based on the Joint Interim Statement (JIS) criteria (18), (i) serum TG ≥150 mg/dL or taking lipid-lowering drugs; (ii) HDL-C <40 mg/dL in men and <50 mg/dL in women, or taking lipid-lowering drugs; (iii) systolic blood pressure (SBP) ≥130 mmHg or diastolic blood pressure (DBP) ≥85 mmHg, or taking antihypertensive drugs; and (iv) fasting blood glucose ≥100 mg/dL or undergoing treatment for diabetes. Participants with < 2 JIS components were considered metabolically healthy, whereas the metabolically unhealthy group included those who met two or more criteria. Since WC is highly correlated with BMI, it was excluded from the definition of metabolically unhealthy status (20).

Subsequently, participants were classified into four groups based on their BMI and metabolic status: (1) metabolically healthy normal weight (MHNW) defined as BMI*<*25kg/m^2^ and healthy metabolic status; (2) metabolically healthy overweight/obese (MHO) defined as BMI ≥ 25 kg/m^2^ and healthy metabolic status; (3) metabolically unhealthy normal weight (MUNW) defined as BMI *<* 25 kg/m^2^ and unhealthy metabolic status; (4) metabolically unhealthy overweight/obese (MUO) defined as BMI ≥ 25 kg/m^2^ and unhealthy metabolic status.

### 2.5 Statistical analysis

The mean and standard deviation were used when the data had a normal distribution, and the median [25th and 75th percentiles] was used when the data had a skewed distribution. Categorical variables were presented as numbers (percentages). Differences in continuous variables were compared using one-way analysis of variance or Kruskal–Wallis one-way analysis of variance. Comparisons between groups were conducted using the chi-squared test or Fisher’s exact test for categorical variables. The relationship between metabolic phenotypes and hypothyroidism development was examined using a multiple logistic regression analysis. A two-tailed P < 0.05 was considered statistically significant. All statistical analyses were performed using Stata version 15.1 statistical software (StataCorp LLC, Texas, USA).

## 3 Results

### 3.1 Baseline characteristics

A total of 3338 subjects with a mean age of 39 ± 12.72 years were included in the present study. Males and females constituted 45.9 and 54.1 percent of the study population, respectively. The MUO (n=1354) phenotype was the most prevalent at baseline. **Table 1** summarizes the baseline characteristics of participants according to their metabolic phenotypes. At baseline, there were significant differences in sex, age, BMI, WC, TC, TG, LDL, HDL, SBP, DBP, FPG, FT4, creatinine (Cr), eGFR, smoking, and physical activity levels among the four groups (P<0.01). However, no differences were observed in TSH and TPO-Ab levels at the start of the study (P>0.05).

**Table 1:** Baseline characteristics of the study population according to different metabolic phenotypes.

Variable		Total (n= 3338)	MHNW (n= 883)	MUNW (n= 423)	MHO (n= 678)	MUO (n= 1354)	*P*-value
Gender, n (%)	Male	1533 (45.9)	399 (45.2)	262 (61.9)	220 (32.4)	652 (48.2)	<0.001
	Female	1805 (54.1)	484 (54.8)	161 (38.1)	458 (67.6)	702 (51.8)	
Age, year		39.00 (12.72)	32.35 (11.66)	41.18 (13.41)	37.59 (10.96)	43.35 (12.00)	<0.001
BMI, kg/m^2^		26.50 (4.53)	21.82 (2.11)	22.97 (1.73)	28.65 (3.25)	29.57 (3.42)	<0.001
WC, cm		87.30 (12.00)	75.47 (7.25)	81.22 (6.92)	90.22 (9.47)	95.44 (9.36)	<0.001
TC, mg/dL		201.37 (42.53)	178.91 (34.53)	202.88 (42.26)	199.06 (35.90)	216.70 (43.76)	<0.001
TG, mg/dL		142.0 (93.0, 205.0)	86.0 (65.0, 111.0)	179.0 (152.75, 232.0)	106.0 (82.0, 133.0)	199.0 (160.0, 259.0)	<0.001
HDL, mg/dL		41.69 (10.98)	47.56 (10.54)	36.40 (8.00)	46.54 (11.34)	37.08 (8.74)	<0.001
LDL, mg/dL		127.69 (35.83)	112.92 (31.37)	127.59 (36.26)	130.03 (31.20)	136.79 (37.61)	<0.001
SBP, mmHg		116.21 (16.47)	107.70 (11.08)	117.06 (16.37)	111.34 (12.34)	123.98 (17.61)	<0.001
DBP, mmHg		76.55 (16.47)	70.79 (8.07)	76.75 (10.41)	74.33 (8.47)	81.37 (10.24)	<0.001
FPG, mg/dL		95.22 (27.74)	86.07 (8.64)	99.43 (35.90)	87.87 (11.76)	103.55 (34.96)	<0.001
TSH, mIU/L		1.54 (1.01, 2.34)	1.55 (1.01, 2.35)	1.48 (0.96, 2.33)	1.61 (1.10, 2.38)	1.51 (0.99, 2.32)	0.175
FT4, ng/dL		1.21 (0.14)	1.24 (0.14)	1.22 (0.14)	1.19 (0.14)	1.19 (0.15)	<0.001
TPO-Ab, IU/mL		5.27 (3.15, 10.31)	5.40 (3.13, 10.45)	5.07 (3.12, 9.55)	5.21 (3.10, 10.61)	5.27 (3.20, 10.30)	0.814
Cr, μmol/L		1.03 (0.15)	1.02 (0.15)	1.06 (0.14)	1.02 (0.14)	1.04 (0.15)	<0.001
eGFR, mL/min/1.73		79.34 (12.23)	84.39 (12.34)	79.54 (12.70)	78.07 (10.90)	76.62 (11.63)	<0.001
Smoking, n (%)	Yes	429 (12.9)	118 (13.4)	70 (16.50)	64 (9.5)	177 (13.1)	0.006
	No	2902 (87.1)	761 (86.6)	353 (83.5)	613 (90.5)	1175 (86.9)	
Physical activity, MET-min/week	Low	2036 (61.4)	546 (62.1)	240 (57.0)	432 (64.1)	818 (60.9)	<0.001
	Moderate	895 (27.0)	247 (28.1)	113 (26.8)	194 (28.8)	341 (25.4)	
	High	386 (11.6)	86 (9.8)	68 (16.2)	48 (7.1)	184 (13.7)	

Continuous variables with normal distribution were reported as mean ± SD; Continuous variables with non-normal distribution were reported as median (interquartile range); Categorical variables were reported as N (%).

* Difference between the groups at baseline, P value is reported based on one-way ANOVA for continuous variables with normal distribution, Kruskal-Wallis for continuous variables with non-normal distribution, and Chi-square for categorical variables.

MHNW, metabolically healthy normal weight; MHO, metabolically healthy overweight/obese; MUNW, metabolically unhealthy normal weight; MUO, metabolically unhealthy overweight/obese; BMI, body mass index; WC, waist circumference; TG, triglycerides; TC, total cholesterol; LDL, low-density lipoprotein-cholesterol; HDL, high-density lipoprotein-cholesterol; FPG, fasting plasma glucose; SBP, systolic blood pressure; DBP, diastolic blood pressure; FT4, free thyroxine; TPO-Ab, thyroid peroxidase antibody; Cr, creatinine; eGFR, estimated glomerular filtration rate.

### 3.2 Association between metabolic phenotypes and the development of hypothyroidism

**Figure 2:**
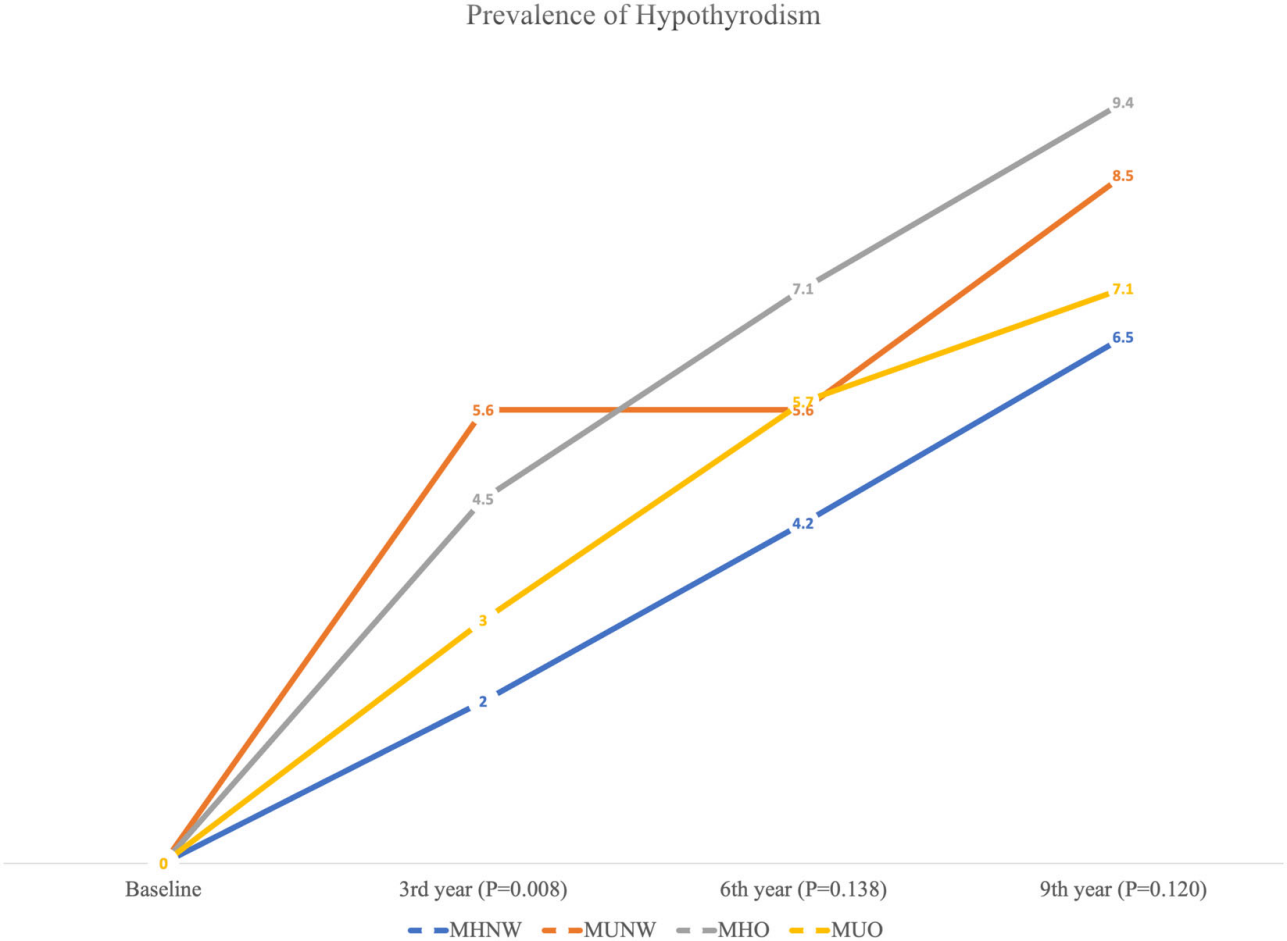
Prevalence of hypothyroidism according to different metabolic phenotypes in the total population.

The frequency (%) of hypothyroidism at each measurement time (baseline and 3^rd^, 6^th^, and 9^th^ year) is provided in **Figure 2** (total population) and **Table 2** (according to male and female participants). In the total population, the chi-square test was only significant (P=0.008) in 3^rd^ year with a higher prevalence of hypothyroidism in the MUNW phenotype followed by MHO, MUO, and MHNW. In later years, the order of prevalence of hypothyroidism (from highest to lowest) was as follows: MHO, MUNW, MUO, and MHNW. However, this difference in the proportion of hypothyroidism was not statistically significant in the 6^th^ (P=0.138) and 9^th^ (P=0.120) years. Moreover, in the 3^rd^ and 9^th^ years of follow-up, hypothyroidism was more prevalent in MUO only in male subjects (P=0.002 and 0.035, respectively).

**Table 2:** Frequency (%) of hypothyroidism according to different metabolic phenotypes in male and female participants.

Hypothyroidism		MHNW	MUNW	MHO	MUO	*P*- value
Male	3^rd^ year	0 (0.0)	9 (4.3)	3 (1.8)	12 (2.2)	0.002^*^
	6^th^ year	4 (1.2)	11 (4.9)	6 (3.1)	20 (3.7)	0.083
	9^th^ year	12 (3.0)	19 (7.3)	6 (2.7)	32 (4.9)	**0.035**
Female	3^rd^ year	13 (3.6)	10 (7.7)	22 (5.8)	22 (3.7)	0.115
	6^th^ year	27 (6.5)	10 (6.8)	37 (8.9)	48 (7.4)	0.593
	9^th^ year	45 (9.4)	17 (10.6)	58 (12.7)	64 (9.2)	0.238

P value is reported based on the Chi-square test (values indicated by ^*^ calculated using Fisher’s Exact test).

MHNW, metabolically healthy normal weight; MHO, metabolically healthy overweight/obese; MUNW, metabolically unhealthy normal weight; MUO, metabolically unhealthy overweight/obese. Values in bold indicates P < 0.05.

**Table 3** shows multiple logistic regression models of the association between hypothyroidism and metabolic profiles in the total population. In the unadjusted model, the MHO phenotype increased the odds of hypothyroidism compared to the MHNW phenotype (OR=1.51; 95% CI=1.04, 2.18; P-value=0.031). Although a higher odds of hypothyroidism was observed for MUNW and MUO phenotypes compared to MHNW, the difference was not statistically significant (P=0.179 and 0.563, respectively). In model 1, only MUNW was significantly associated with hypothyroidism compared to the MHNW phenotype after adjustment for the effect of age and sex (OR=1.70; 95% CI=1.08, 2.67; P-value=0.022). The association between MHO and hypothyroidism was not significant in model 1 (P=0.062). Similarly, in model 2, the odds of hypothyroidism was higher in the MUNW phenotype than in the MHNW phenotype after adjusting for the effects of age, sex, and TPO-Ab (OR=1.76; 95% CI=1.12, 2.79; P-value=0.015). In model 3, the effects of waist circumference, Cr, smoking, and physical activity were adjusted in addition to the previous variables. In this model, the odds of hypothyroidism were higher in MUNW (OR=1.86; 95% CI=1.17, 2.96; P-value=0.008), MHO (OR=1.71; 95% CI=1.09, 2.67; P-value=0.018), and MUO (OR=1.64; 95% CI=1.03, 2.62; P-value=0.036) phenotypes than in MHNW.

**Table 3:** Results of multiple logistic regression for developing hypothyroidism based on different obesity phenotypes during 9 years of follow-up in participants.

		Unadjusted model		Model 1		Model 2		Model 3	
		OR (95% CI)	*P*	OR (95% CI)	*P*	OR (95% CI)	*P*	OR (95% CI)	*P*
***Total***	MHNW	Ref.		Ref.		Ref.		Ref.	
	MUNW	1.35 (0.87, 2.08)	0.179	1.70 (1.08, 2.67)	**0.022**	1.76 (1.12, 2.79)	**0.015**	1.86 (1.17, 2.96)	**0.008**
	MHO	1.51 (1.04, 2.18)	**0.031**	1.44 (0.98, 2.10)	0.062	1.38 (0.94, 2.04)	0.102	1.71 (1.09, 2.67)	**0.018**
	MUO	1.10 (0.79, 1.55)	0.563	1.22 (0.84, 1.76)	0.302	1.24 (0.85, 1.81)	0.258	1.64 (1.03, 2.62)	**0.036**

Model 1: adjusted for age and gender.

Model 2: adjusted for age, gender, TPO-Ab.

Model 3: adjusted for age, gender, TPO-Ab, waist circumference, Cr, smoking, and physical activity.

MHNW, metabolically healthy normal weight; MHO, metabolically healthy overweight/obese; MUNW, metabolically unhealthy normal weight; MUO, metabolically unhealthy overweight/obese. Values in bold indicates P < 0.05.

**Table 4** shows the results of multiple logistic regression analysis of the association between metabolic phenotypes and hypothyroidism according to sex. In the unadjusted model, the MUNW phenotype increased the risk of hypothyroidism compared to MHNW only in males (OR=2.53; 95% CI=1.21, 5.31; P-value=0.014). This association remained significant after adjusting for the effect of age in model 1 (P=0.019), age and TPO-Ab in model 2 (P=0.028), and age, TPO-Ab, waist circumference, Cr, smoking, and physical activity in model 3 (P=0.034). Moreover, in females, the MHO phenotype increased the risk of hypothyroidism compared to MHNW in models 1 (OR=1.56; 95% CI=1.02, 2.39; P-value=0.039) and 3 (OR=1.87; 95% CI=1.15, 3.05; P-value=0.012).

**Table 4:** Results of multiple logistic regression for developing hypothyroidism based on different obesity phenotypes during 9 years of follow-up according to gender.

		Unadjusted model		Model 1		Model 2		Model 3	
		OR (95% CI)	*P*	OR (95% CI)	*P*	OR (95% CI)	*P*	OR (95% CI)	*P*
***Male***	MHNW	Ref.		Ref.		Ref.		Ref.	
	MUNW	2.53 (1.21, 5.31)	**0.014**	2.45 (1.16, 5.19)	**0.019**	2.38 (1.10, 5.14)	**0.028**	2.35 (1.07, 5.16)	**0.034**
	MHO	0.90 (0.33, 2.44)	0.843	0.88 (0.32, 2.38)	0.802	0.95 (0.35, 2.62)	0.929	0.87 (0.27, 2.77)	0.810
	MUO	1.66 (0.85, 3.27)	0.139	1.51 (0.75, 3.03)	0.244	1.52 (0.75, 3.11)	0.246	1.36 (0.52, 3.59)	0.534
***Female***	MHNW	Ref.		Ref.		Ref.		Ref.	
	MUNW	1.14 (0.63, 2.06)	0.655	1.35 (0.73, 2.48)	0.333	1.46 (0.79, 2.69)	0.227	1.51 (0.81, 2.79)	0.191
	MHO	1.40 (0.93, 2.12)	0.106	1.56 (1.02, 2.39)	**0.039**	1.50 (0.98, 2.32)	0.063	1.87 (1.15, 3.05)	**0.012**
	MUO	0.98 (0.65, 1.46)	0.914	1.18 (0.76, 1.86)	0.460	1.21 (0.77, 1.91)	0.401	1.65 (0.96, 2.83)	0.070

Model 1: adjusted for age.

Model 2: adjusted for age and TPO-Ab.

Model 3: adjusted for age, TPO-Ab, waist circumference, Cr, smoking, and physical activity.

MHNW, metabolically healthy normal weight; MHO, metabolically healthy overweight/obese; MUNW, metabolically unhealthy normal weight; MUO, metabolically unhealthy overweight/obese. Values in bold indicates P < 0.05.

## 4 Discussion

This study examined the relationship between obesity phenotypes and the incidence of hypothyroidism, focusing on differences between males and females. The present cohort study demonstrated a higher prevalence of hypothyroidism in the MUNW phenotype followed by MHO, MUO, and MHNW in 3^rd^ year of follow-up, and in the 3^rd^ and 9^th^ years of follow-up, hypothyroidism was more prevalent among MUO only in males. Males with MUO, MHO, and MUNW phenotypes had a higher risk of hypothyroidism than those with MHNW phenotypes. In females, the MHO phenotype increased the risk of hypothyroidism compared with MHNW.

In a longitudinal study conducted in Shandong, China (21), the authors reported that the non-MHNO group had a significantly higher incidence of hypothyroidism than the MHNO group in males, and the MHO, MUNO, and MUO phenotypes were independent risk factors for the development of hypothyroidism compared with the MHNO phenotype in males but not in females. Previously, in Tehran, Iran (15), researchers explored the relationship between thyroid function and obesity phenotype development, which was different from our goal.

It is currently unclear whether obesity is associated with thyroid autoimmunity (8). A previous study on subjects without thyroid autoimmunity at baseline found that the abdominal obesity phenotype had no significant impact on the development of TPO-Ab positivity over time (22). The results of a prospective cohort study indicated a higher likelihood of developing subclinical hypothyroidism in participants with metabolic syndrome (MetS) at baseline (10). Few studies have examined the association between thyroid autoimmunity and MetS. A cross-sectional study reported that thyroid autoimmunity was associated with high glycated hemoglobin levels, central obesity, dyslipidemia, and MetS among euthyroid individuals (23). In contrast, a study on postmenopausal euthyroid women found no association between TPOAb positivity and MetS prevalence (24). In another cross-sectional study (25), using KNHANES VI data of 4775 euthyroid subjects, the researchers found that thyroid autoimmunity is associated with poor physiological health outcomes, such as abdominal obesity, low HDL cholesterol, and hypertension. There could be a number of factors contributing to such inconsistent results, such as ethnicity, diet, lifestyle, age, and sex composition, among others. Therefore, anthropometric state and metabolic disorders are likely linked to hypothyroidism.

Despite the lack of a complete understanding of the mechanisms underlying obesity phenotypes and hypothyroidism, some explanations have been suggested. Chronic low-grade inflammation has been observed in obese individuals. An increase in inflammatory cytokines, including IL-1, IL-6, and TNF-α, inhibits sodium-iodide symporter (NIS) expression, influences iodide uptake activity, and affects thyroid morphology (26, 27). Leptin may also suppress TSH-induced thyroid function in obese individuals (28). The deiodinase enzyme may also be modulated by chronic inflammation and may affect thyroid function (29, 30). It is also possible that lipotoxicity affects the thyroid (31, 32). According to a recent study, palmitic acid reduces thyroid hormone synthesis by downregulating the expression and activity of NIS, thyroglobulin, and thyroperoxidase (31). Hypothyroidism caused by high-fat diet may be caused by endoplasmic reticulum stress (32). There are differences between men and women in the association between obesity/metabolic disorders and thyroid diseases (33, 34). According to a cohort study, obesity and metabolic conditions may influence thyroid cancer development differently depending on sex (34).

Our study found that males with MHO, MUNO, and MUO phenotypes were independently at risk for hypothyroidism, while females were not. We found a sex difference in the association between obesity phenotypes and hypothyroidism, although the mechanisms underlying this association are unclear. First, testosterone and estradiol affect thyroid function in different ways (35, 36). Second, visceral adipose tissue accumulates more commonly in men than in women, indicating that obesity poses a greater threat to men (37). The risk of hypothyroidism may vary depending on the obesity phenotype, which may result in sex-specific alterations in sex hormones. We found that normal weight and a healthy metabolic state reduced the risk of hypothyroidism in both men and women. The validation of our findings and elucidation of the underlying mechanisms require further research.

This study has several limitations that should be considered when interpreting the findings. Owing to its observational nature, this cohort study could not infer causal relationships. A bidirectional and complex relationship exists between obesity and thyroid function (27). Thyroid organs may be susceptible to lipotoxicity (31, 32), and thyroid hormones play a role in the metabolic control of glucose and lipids (38). Although we conducted a cohort study with baseline euthyroid participants and adjusted for confounding factors, we cannot exclude reverse causation and unmeasured confounders. Second, our study used BMI as a measure to diagnose obesity. The association between obesity phenotypes and hypothyroidism may be better understood if further studies are performed using body composition and WC data. Thyroid function measurements were performed at each visit to diagnose hypothyroidism. Finally, this cohort included subjects from Tehran, Iran, who underwent regular health examinations. Further research is needed to determine whether our findings apply to other populations with different characteristics.

It is important to note, however, that our study has several strengths despite its limitations. First, this is the first cohort study to examine the sex-specific relationship between obesity phenotypes and hypothyroidism in Iran. Second, our study found that not only the MUO phenotype was an independent risk factor for hypothyroidism in males, but also the MHO and MUNO phenotypes, providing insight into hypothyroidism risk factors in men. As hypothyroidism is more common in females, less attention has been paid to males in the past. Clinical practice should focus on males with unhealthy metabolic phenotypes because they are more likely to develop hypothyroidism.

## 5 Conclusion

Our findings in the TTS cohort showed that obesity and metabolic abnormalities were related to an elevated risk of hypothyroidism, particularly in males. We found that obesity phenotypes and hypothyroidism were not associated in females, in contrast to the findings in males. The results of this study highlight sex differences in the association between metabolic phenotypes and the risk of hypothyroidism in a baseline euthyroid population. Males with unhealthy metabolic phenotypes should be given special attention. Further research is needed to verify and identify the possible mechanisms explaining the sex differences in this association.

## Data availability statement

The original contributions presented in the study are included in the article/supplementary material. Further inquiries can be directed to the corresponding author.

## Ethics statement

The studies involving human participants were reviewed and approved by Research Institute for Endocrine Sciences (RIES) of Shahid Beheshti University of Medical Sciences (code: IR.SBMU.ENDOCRINE.REC.1400.116). The patients/participants provided their written informed consent to participate in this study.

## Author contributions

BA, and MV contributed to the design of study, conducted the searches, drafted and edited the manuscript. ARA and MM contributed to the design of the study, analyzing the data, and revised the manuscript. FH, AA and MV advised and revised the manuscript. All authors have read and approved the final version of the manuscript. MV has primary responsibility for final content. All authors contributed to the article and approved the submitted version.
